# Assessment of Epidemiology Capacity in State Health Departments — United States, 2017

**DOI:** 10.15585/mmwr.mm6733a5

**Published:** 2018-08-24

**Authors:** Jessica Arrazola, Nancy Binkin, Maria Israel, Aaron Fleischauer, Elizabeth R. Daly, Robert Harrison, Jeffrey Engel

**Affiliations:** ^1^Council of State and Territorial Epidemiologists, Atlanta, Georgia; ^2^University of California, San Diego School of Medicine, La Jolla, California; ^3^Office of Public Health Preparedness and Response, CDC; ^4^North Carolina Division of Public Health; ^5^New Hampshire Department of Health and Human Services; ^6^California Department of Public Health.

## Abstract

In 2017, the Council of State and Territorial Epidemiologists performed its sixth periodic Epidemiology Capacity Assessment, a national assessment that evaluates trends in workforce size, funding, and epidemiology capacity among state health departments. A standardized web-based questionnaire was sent to the state epidemiologist in the 50 states, the District of Columbia (DC), and the U.S. territories and the Federated States of Micronesia inquiring about the number of current and optimal epidemiologist positions; sources of epidemiology activity and personnel funding; and each department’s self-perceived capacity to lead activities, provide subject matter expertise, and obtain and manage resources for the four Essential Public Health Services (EPHS)* most closely linked to epidemiology. From 2013 to 2017, the number of state health department epidemiologists^†^ increased 22%, from 2,752 to 3,369, the greatest number of workers since the first full Epidemiology Capacity Assessment enumeration in 2004. The federal government provided most (77%) of the funding for epidemiologic activities and personnel. Substantial to full capacity (50%–100%) was highest for investigating health problems (92% of health departments) and monitoring health status (84%), whereas capacity for evaluating effectiveness (39%) and applied research (29%) was considerably lower. An estimated additional 1,200 epidemiologists are needed to reach full capacity to conduct the four EPHS. Additional resources might be needed to ensure that state health department epidemiologists possess the specialized skills to deliver EPHS, particularly in evaluation and applied epidemiologic research.

Epidemiology Capacity Assessments were conducted in 2001, 2004, 2006, 2009, and 2013, with supplementary workforce enumeration conducted in 2010. Since 2004, 100% of the states and DC have responded to the assessment. The Epidemiology Capacity Assessment was updated in 2017 to reflect expansion of health department programs into genomics, informatics, and vital statistics. A core set of questions has remained essentially unchanged and permits the monitoring of trends in the epidemiology workforce employed by the 50 states, DC, and U.S. territories; current funding sources for epidemiology activities and personnel; capacity in the four EPHS relevant to epidemiology (*1*); and issues in hiring, training, and retaining skilled epidemiologists to meet current needs and changing priorities.

After the council piloted the instrument, the 2017 Epidemiology Capacity Assessment was disseminated electronically to state and territorial epidemiologists using Qualtrics,^§^ an online survey tool. Data collection began April 28, 2017, and was completed August 11, 2017. Virtual technical assistance was provided to support completion of the Epidemiology Capacity Assessment. All 50 states, DC, and three territories responded to the assessment; this analysis includes responses from U.S. states and DC. The number of full-time equivalent (FTE) epidemiologist positions (to the nearest 0.1 FTE) was collected by program area and source of funding. Respondents subjectively evaluated their capacity for each EPHS as none (0%), minimal (1%–24%), partial (25%–49%), substantial (50%–74%), almost full (75%–99%), and full (100%). For each program area, jurisdictions were asked to provide an overall judgement of capacity^¶^ to meet all four EPHS.

A total of 3,369 FTE epidemiologist positions were enumerated in 2017, a 22% increase over the 2,752 reported in 2013. Overall, the number of epidemiologists per 100,000 population was 1.04 (range = 0.2–5.6), 20% higher than the 0.87 per 100,000 calculated in 2013. The size of the epidemiology workforce in each state ranged from five to 208.

The federal government provided 77% of funding for epidemiologic activities and personnel in 2017, a slight decrease from 79% in 2013. State governments provided an additional 19%, an amount unchanged since 2013, and the remaining 4% came from other sources. CDC was the source of 89% of the 2017 federal funding for epidemiology personnel.

Among program areas, infectious diseases accounted for 1,838 (55%) of the 3,369 epidemiology positions, followed by maternal and child health (MCH) (10%) and chronic diseases (9%) (Table) (Figure 1). Program areas with the fewest epidemiologists included substance abuse, occupational health, oral health, mental health, and genomics. The number of infectious disease positions has increased steadily since program area positions were first measured in 2004; infectious disease positions experienced the largest absolute increase from 2013 to 2017, with the addition of 487 positions (Figure 1). In contrast, the number of epidemiologists in preparedness (formerly bioterrorism and emergency response) positions has been declining since 2004, and the decline was steeper (-55%) during 2013–2017. The number of MCH epidemiologists has gradually increased, and the number of injury epidemiologists, after experiencing a gradual decline, is higher than any time in the past. The number of chronic disease, and environmental, occupational, and oral health program epidemiologists has remained stable or declined since 2004.

**TABLE:** Epidemiology full-time equivalents (FTEs), by program area — Council of State and Territorial Epidemiologists Epidemiology Capacity Assessment, 50 states and the District of Columbia, 2017

Program area	FTEs currently filled (% of total)	Additional FTEs needed	Optimal* (% of ideal FTEs currently met)^†^	Vacant positions^§^	Positions actively being recruited^¶^
Infectious disease	1,838.2 (54.6)	338.4	2,176.6 (84.4)	158.6	140.6
Maternal and child health	321.2 (9.5)	122.0	443.2 (72.4)	44.7	37.7
Chronic disease	304.4 (9.0)	136.6	441.0 (69.0)	41.7	36.7
Environmental health	221.7 (6.6)	121.9	343.6 (64.5)	23.3	18.3
Informatics	95.7 (2.8)	91.2	186.9 (51.2)	15.0	14.0
Vital statistics	110.7 (3.3)	62.0	172.7 (64.1)	13.2	13.2
Injury	102.5 (3.0)	56.9	159.4 (64.3)	11.2	13.2
Preparedness	117.6 (3.5)	35.7	153.3 (76.7)	9.5	10.5
Substance abuse	58.6 (1.7)	63.7	122.3 (47.9)	8.8	6.3
Occupational health	28.4 (0.8)	38.1	66.5 (42.7)	7.5	5.5
Mental health	4.0 (0.1)	42.3	46.3 (8.6)	6.0	6.0
Oral health	18.0 (0.5)	25.0	43.0 (41.9)	3.0	2.0
Genomics	4.4 (0.1)	20.2	24.6 (17.9)	1.3	3.3
Other	143.4 (4.3)	45.1	188.5 (76.1)	9.6	6.6
**Total**	**3,368.8 (100.0)**	**1,199.1**	**4,567.9 (73.7)**	**353.4**	**313.9**

* Currently filled plus additional needed.

^†^ Currently filled/ideal x 100.

^§^ Positions to be filled at a state health department for which work is available and the job could start within 30 days.

^¶^ Vacant positions human resources working actively to fill.

**FIGURE 1:**
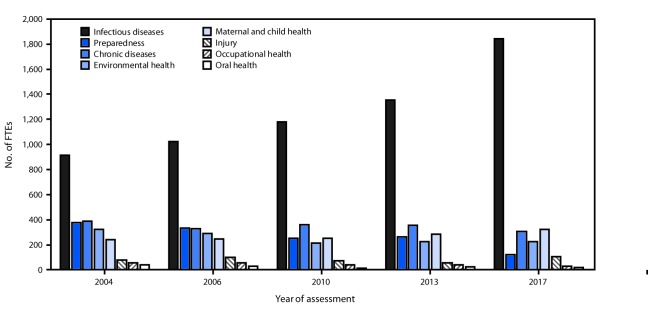
Epidemiology full-time equivalents (FTEs), by program area* — Council of State and Territorial Epidemiologists Epidemiology Capacity Assessment, United States, 2004–2017 * Preparedness was formerly bioterrorism.

Participating state epidemiologists expressed the need for nearly 1,200 additional epidemiologists to reach full capacity to provide the four EPHS, a 36% increase over current levels (Table). Nearly 600 of these additional needed epidemiologists are in the areas of infectious diseases, MCH, and chronic diseases, areas which already represent 75% of the epidemiology workforce. Although jurisdictions reported the need for additional positions in programs for substance abuse (64), mental health (42), and genomics (20), these program areas accounted for only 4% of the optimal total positions (those currently filled plus those needed). At the time of the assessment, among 353 vacancies nationwide, 314 (89%) positions were being actively recruited, including 141 (45%) in infectious disease program areas.

In 2017, 84% and 92% of jurisdictions perceived that they had substantial-to-full capacity for monitoring health status (EPHS #1) and investigating health problems and hazards (EPHS #2), respectively, similar to responses in 2013 (82% and 90%, respectively). In contrast, 39% of the 51 reporting jurisdictions reported substantial-to-full capacity for evaluation of effectiveness (EPHS #9), up from 35% in 2017, and 22% reported similar capacity for research (EPHS #10), compared with 29% in 2013.

When overall capacity was examined by program area, substantial-to-full capacity was highest for infectious diseases, chronic diseases, and MCH and was lowest for genomics, mental health, and substance abuse (Figure 2). From 2013 to 2017, substantial-to-full capacity changed by <5 percentage points for all program areas, with the exception of chronic diseases (increase from 66% to 78%), environmental health (decline from 49% to 43%), and mental health (decline from 8% to 2%). Preparedness, which experienced a 55% decrease in the number of epidemiologists, reported a decline in capacity of two percentage points, from 69% to 67%.

**FIGURE 2:**
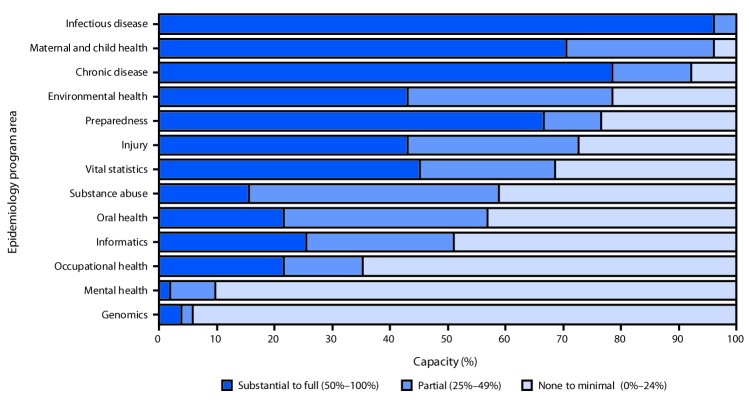
Overall current epidemiologic capacity to provide four Essential Public Health Services* — Council of State and Territorial Epidemiologists Epidemiology Capacity Assessment, United States, 2017 * The four Essential Public Health Services (EPHS) capacities evaluated included 1) monitoring health status to identify and solve community health problems (EPHS #1); 2) diagnosing and investigating health problems and health hazards in the community (EPHS #2); 3) evaluating effectiveness, accessibility, and quality of personal and population-based health services (EPHS #9); and 4) researching new insights and innovative solutions to health problems (EPHS #10).

## Discussion

Overall, the 2017 Epidemiology Capacity Assessment documented that, although the epidemiology workforce continues to grow, there is an ongoing unmet need for additional epidemiologist positions in well-established areas, such as infectious diseases, and in emerging areas, including substance abuse, mental health, and informatics. Whereas capacity is high in monitoring health status and in diagnosing public health problems, capacity in evaluation and research lags behind, and no strict correlation exists between growth in workforce size and EPHS capacity. Program area capacity is high in well-established areas but is lower for newer areas such as genomics and informatics and for areas with low and waning numbers of epidemiologists, such as oral health and environmental health.

The recent increase in infectious disease and injury positions and decrease in preparedness positions might reflect changes in funding sources and priorities. In the past 2 decades, the Epidemiology and Laboratory Capacity and Public Health Emergency Preparedness cooperative agreements have provided funding to health departments for many infectious disease and preparedness epidemiology positions (*2*,*3*) in response to emerging and reemerging threats (*4*,*5*). However, funding recently has decreased for preparedness (*6*) and increased for infectious diseases, and some epidemiologists previously working in preparedness might have shifted to infectious disease positions. Such a shift might explain why capacity in preparedness has not decreased substantially in the face of the 55% decrease in preparedness positions. Recently, CDC has also increased funding to injury programs in response to the U.S. opioid epidemic through cooperative agreements for the Prevention for States program (*7*), Data-Driven Prevention Initiative (*8*), and Enhanced State Opioid Overdose Surveillance (*9*).

The findings in this report are subject to at least two limitations. First, the number of epidemiology positions is measured only for state health departments and does not include epidemiologists working in other state agencies such as occupational health epidemiologists working in state departments of labor. Second, the data on public health capacity are subjective, although when the analyses were limited to those jurisdictions with the same state epidemiologist in 2013 and 2017, EPHS capacity findings were essentially unchanged.

Despite the increase in the number of epidemiologists since 2013, only infectious diseases, preparedness, chronic diseases, and MCH have substantial-to-full capacity to conduct EPHS. Serious capacity deficits remain, especially in areas of substance abuse, mental health, occupational health, environmental health, and informatics at a time when these areas are assuming increasing importance.** Capacity in evaluation and research is particularly low. The increase in program area capacity that accompanied the increase in epidemiologists from 2009 to 2013 did not continue from 2013 to 2017. The findings suggest that hiring alone, without considering the specialized skills needed to improve the current perceived gaps in capacity, might no longer result in capacity improvements. Gaps in capacity affect the ability of public health agencies to respond and leave them vulnerable to emerging threats such as the current opioid epidemic. Hiring epidemiologists with evaluation and research skills or retraining existing staff members and prioritizing these skills in state health departments might help achieve full EPHS capacity.

**Summary**

**What is already known about this topic?**

Overall, the state health department epidemiology workforce has increased over time, but an unmet need remains high. Evaluation and research capacity has improved but remains low. Most funding has come from the federal government.

**What is added by this report?**

From 2013 to 2017, the number of state epidemiologists increased by 22%. Several emerging program areas remain seriously understaffed. The federal government continues to fund most (77%) state epidemiology activities and personnel. Capacity in four assessed Essential Public Health Services has remained stable or has declined in all areas except evaluation.

**What are the implications for public health practice?**

More epidemiologists and greater expertise in evaluation and applied research are needed to achieve comprehensive health department capacity.

## Footnotes

* The four EPHS capacities evaluated in the assessment included 1) monitoring health status to identify and solve community health problems (EPHS #1); 2) diagnosing and investigating health problems and health hazards in the community (EPHS #2); 3) evaluating effectiveness, accessibility, and quality of personal and population-based health services (EPHS #9); and 4) researching new insights and innovative solutions to health problems (EPHS #10).

§ https://www.qualtrics.com/.

¶ For purposes of the Epidemiology Capacity Assessment, capacity was defined as “the state health department’s ability to lead activities, provide subject matter expertise, and apply for, receive, and manage resources to conduct key activities.”

** https://www.cdc.gov/about/organization/strategic-framework/index.html.
