# *Erratum:* Vol. 67, No. 33

**DOI:** 10.15585/mmwr.mm6816a4

**Published:** 2019-04-26

In the report “Assessment of Epidemiology Capacity in State Health Departments — United States, 2017,” on page 936, a Table contained errors. The corrected Table is below.

**TABLE:** Epidemiology full-time equivalents (FTEs), by program area — Council of State and Territorial Epidemiologists Epidemiology Capacity Assessment, 50 states and the District of Columbia, 2017

Program area	FTEs currently filled (% of total)	Additional FTEs needed	Optimal* (% of ideal FTEs currently met)^†^	Vacant positions^§^	Positions actively being recruited^¶^
Infectious disease	1,838.2 (54.6)	338.4	2,176.6 (84.4)	158.6	140.6
Maternal and child health	321.2 (9.5)	122.0	443.2 (72.4)	**41.7**	**36.7**
Chronic disease	304.4 (9.0)	136.6	441.0 (69.0)	**44.7**	**37.7**
Environmental health	221.7 (6.6)	121.9	343.6 (64.5)	23.3	18.3
Informatics	95.7 (2.8)	91.2	186.9 (51.2)	**11.2**	**13.2**
Vital statistics	110.7 (3.3)	62.0	172.7 (64.1)	**15.0**	**14.0**
Injury	102.5 (3.0)	56.9	159.4 (64.3)	**9.5**	**10.5**
Preparedness	117.6 (3.5)	35.7	153.3 (76.7)	**13.2**	**13.2**
Substance abuse	58.6 (1.7)	63.7	122.3 (47.9)	8.8	6.3
Occupational health	28.4 (0.8)	38.1	66.5 (42.7)	**3.0**	**2.0**
Mental health	4.0 (0.1)	42.3	46.3 (8.6)	**1.3**	**3.3**
Oral health	18.0 (0.5)	25.0	43.0 (41.9)	**7.5**	**5.5**
Genomics	4.4 (0.1)	20.2	24.6 (17.9)	**6.0**	**6.0**
Other	143.4 (4.3)	45.1	188.5 (76.1)	9.6	6.6
**Total**	**3,368.8 (100.0)**	**1,199.1**	**4,567.9 (73.7)**	**353.4**	**313.9**

* Currently filled plus additional needed.

^†^ Currently filled/ideal x 100.

^§^ Positions to be filled at a state health department for which work is available and the job could start within 30 days.

^¶^ Vacant positions human resources working actively to fill.

